# Body Fat Percentage and Normal-Weight Obesity in the Chinese Population: Development of a Simple Evaluation Indicator Using Anthropometric Measurements

**DOI:** 10.3390/ijerph19074238

**Published:** 2022-04-01

**Authors:** Yuetong Zhu, Zimin Wang, Hitoshi Maruyama, Ko Onoda, Qiuchen Huang

**Affiliations:** 1 Graduate School of Health and Welfare Sciences, International University of Health and Welfare, Tochigi 324-8501, Japan; zhuyuetong2018@gmail.com; 2 Human Health Sciences, Graduate School of Medicine, Kyoto University, Kyoto 606-8397, Japan; 3 Department of Physical Therapy, School of Health Science, International University of Health and Welfare, Tochigi 324-8501, Japan; hmaru@iuhw.ac.jp (H.M.); ko_onoda@iuhw.ac.jp (K.O.); 4 Department of Physical Therapy, School of Rehabilitation Medicine, Capital Medical University, Beijing 100069, China; qiuchen_1984@126.com

**Keywords:** body circumferences, body fat percentage, (hip + waist)/height ratio, obesity

## Abstract

Few studies explore the associations between body fat percentage (BFP) prediction and evaluation indicators for Chinese with normal-weight obesity. We aimed to explore convenient and cost-free BFP evaluation indicators to routinely monitor BFP status in Chinese patients with normal-weight obesity. Participants (N = 164) were divided into three groups according to body mass index (BMI) and BFP: normal-weight lean, normal-weight obese, and overweight and obese. Differences in body composition and circumference were compared to examine the relationship between BFP and circumference, determine a simple evaluation indicator reflecting BFP, and identify cutoff values for normal-weight obesity circumference. Significant differences in body composition and circumference were observed among the three groups. The correlation between thigh/height, hip/height, (hip + waist)/height, and BFP was stronger than that with BMI. The (hip + waist)/height ratio was the indicator most reflective of BFP (95% confidence interval: 3.004–9.018, *p* = 0.013), and a ratio above 1.115 (95% confidence interval: 0.936–0.992, *p* < 0.001) was predictive of normal-weight obesity. Furthermore, we suggest that the upper value for a normal BMI in Chinese individuals be lowered to 23.4 kg/m^2^ (95% confidence interval: 0.984–0.999, *p* < 0.001). The (hip + waist)/height ratio can be used with body mass index for a more accurate evaluations of BFP abnormalities and health risks.

## 1. Introduction

Obesity has become a major public health concern. In 2019, according to epidemiological data, the prevalence of overweight and obesity in Chinese adults was 34.3% and 16.4%, respectively. In recent years, Chinese dietary patterns have changed dramatically, along with a decline in physical activity, resulting in more than half of the adult Chinese population being overweight or obese [1]. The average body mass index (BMI) of Chinese individuals increased from 22.7 kg/m^2^ in 2004 to 24.4 kg/m^2^ in 2018 [2]. However, owing to the complex relationship between individual-level risk factors, an increasing number of studies advocate that body fat percentage (BFP) is a better indicator of obesity than BMI, as BMI does not consider body composition [3]. 

BMI is a simple formula based only on height and weight; however, bodyweight comprises fat, muscle, bone, and water. A considerable proportion of people with a normal BMI (18.5–24.9 kg/m^2^) have metabolic syndrome, and BFP combined with BMI may be useful risk assessment indicators of metabolic disease [4]. Meanwhile, the sensitivity of BMI has proven to be low, with more than half (51%) of the patients with abnormal BFP not identified as obese using BMI [5]. BMI should not be considered the only criterion for obesity, particularly in patients with a BMI of less than 30 kg/m^2^ [6]. From as early as 2000, the World Health Organization has recognized the discrepancy between BMI and BFP in the Asian population, recommending that the threshold BMI for obesity in Asians be reduced to 27.5 kg/m^2^ [7].

Since the declaration of the coronavirus disease 2019 (COVID-19) pandemic, the lifestyle and eating habits of people worldwide have been affected and has placed them at a greater risk of obesity. Living with stress during the COVID-19 pandemic may also have consequences on health. Stress increases food consumption, raises interest in highly palatable foods, increases emotional instability, and worsens quality of life [8]. During the pandemic, people gained weight as physical activity declined significantly, coupled with an increase in screen viewing and sitting time This sedentary lifestyle may destabilize or exacerbate hyperglycemia and hypertension [9]. People should be monitored during the COVID-19 pandemic to prevent obesity and maintain their general wellbeing [10]. A reasonable diet and appropriate home exercise are recommended, and the development of training methods and evaluation tools for home exercises during COVID-19 lockdowns is a necessity [11].

In 2000, WHO/IASO/IOTF lowered the BMI range in Asians, and the cut-offs for overweight (≥23.0 kg/m^2^) and obesity (25.0 kg/m^2^). However, these cut-offs were obtained from data on Chinese people living in Southeast Asia or southern China [12]. Differences in the relationship between BFP and BMI could be observed since the northern Chinese (Beijing) have bigger body builds than the southern Chinese [13,14]. Lowering cutoff values by three units has implications for northern Chinese and Japanese people [15]. The WHO/IASO/IOTF meeting did not become official WHO policy for transmission to governments. On the basis of the BMI range defined by the WHO, the Japanese Society for the Study of Obesity proposed Japanese-specific BMI cut-off points that have since been accepted as the standard classification for obesity [16], but still considering 25 kg/m^2^ as an indicator of health risk. This is similar in China, where a proportion of Asian people with a high risk of type 2 diabetes and cardiovascular disease is substantial at the BMI cut-off point (25 kg/m^2^) [17].

Recently, the concept of normal-weight obesity (NWO) has been introduced, in which BMI is normal, but BFP is abnormally high. Patients with NWO have a greater risk of cardiac and metabolic diseases, atherosclerosis, impaired cardiac function, and higher mortality [18]. The risk of metabolic syndrome in patients with NWO is four times higher than in patients considered to be normal-weight lean (NWL). NWL is defined as a BMI in the range of 18.5–24.9 kg/m^2^, with no abnormal body fat accumulation because adipose tissue mainly accumulates in the trunk [19].

Body fat distribution varies across races. Asians are more likely to accumulate abdominal fat even with a lower BMI. In a comparison of multiethnic populations, Asians had the highest BFP for the same BMI [20]. Therefore, a more appropriate evaluation of obesity should be based on BFP, which can be measured using various instruments. Bioelectrical impedance analysis (BIA) is the fastest, cheapest, and easiest method for body composition evaluation [21]. However, access to body composition assessment machines is limited and particularly difficult for people living in impoverished areas due to the high cost of these technologies. Thus, it is important to measure BFP using simple and cost-effective methods, such as anthropometry. The reproducibility of anthropometry is high, and measurement errors due to the anatomical location have no effect on the prediction of cardiovascular and diabetic risk factors [22].

Most current anthropometry studies focus on waist circumference (WC), waist-to-height ratio (WHtR), and waist-to-hip ratio (WHR) as indicators of obesity. Studies have shown that WC plays a major role in reflecting BFP levels [23]. WHtR and WHR are also superior to BMI and provide an improved estimate of cardiovascular risk factors. The World Health Organization also encourages the additional use of WC or WHR to evaluate overweight or obesity [24].

While WHtR is becoming increasingly popular, it does not appear to be better than WC when estimating total abdominal fat mass [25]. The use of WC as a predictor of abdominal fat mass has also been criticized because of its low correlation coefficient [10]. A pilot study found that the WC–height ratio may be a better predictor of cardiovascular disease risk than WC, as WC is not adjusted for height normalization. Moreover, the WHR is not a good predictor of obesity, as the changes in the waist and hip circumference are relative and simultaneous, and even effective weight loss may not result in significant changes in the WHR [26]. The use of anthropometric indicators of only one area to assess BFP is limited, as differences in fat distribution may interfere with the accuracy of the assessment. It may be more beneficial and accurate to assess BFP using the circumference ratios of multiple sites.

Currently, studies have shown that body circumference, including wrist, upper arm, neck, waist, hip, thigh, and calf circumference, can be used to assess obesity, but whether the ratio between circumference can still be used as an assessment of obesity remains unclear [27,28,29,30]. Moreover, the ethnicity and age span of participants in the available studies are large. The most suitable circumference for BFP evaluation in Asians remains unknown. Establishing prediction formulas for different races to accurately estimate adiposity would be of great clinical importance. Thus, this study aimed to assess body composition using BIA to help determine a suitable circumference for daily BFP monitoring and simple early screening of NWO. The hypothesis of this study is that the use of anthropometric measurements combined with BMI to increase the screening accuracy of NWO is a convenient and cost-effective method for the prediction of BFP.

## 2. Materials and Methods

### 2.1. Participants

All participants signed a written informed consent form for data collection after fully understanding the purpose and procedures of the study. Data collection was performed from 15 July to 20, 2021 at a community center in Beijing. The study was approved by the ethics committee of the relevant institution. Overall, 164 healthy residents of Beijing, China were enrolled in this study. In total, 40 (24.39%) were males and 124 (75.61%) were females. Their mean age was 55.6 ± 8.5 years, and the mean BMI was 25.1 ± 3.2 kg/m^2^. Only participants who were healthy and living independently were included in the study. Exclusion criteria were pregnancy, central nervous system diseases, psychosomatic diseases, inability to walk independently or balance problems, or the presence of any extracorporeal devices (such as a pacemaker). 

### 2.2. Anthropometric Measurements

The body measurements were made using an anthropometric measuring tape (Cescorf Corp., Porto Alegre, Brazil). Anthropometry data were collected by a physical therapist with at least 5 years of clinical experience, and who has concurrent physiotherapist practitioner qualifications in both Japan and China. The percentage technical error of measurement (%TEM) of all analyzed variables was within an internationally acceptable standard of ≤1.5% [31]. Participants underwent detailed anthropometric assessments of the body, including wrist, upper arm, neck, waist, hip, thigh, and calf circumference (Table 1). All measurements except waist measurements were conducted according to the standard protocol of the International Society for the Advancement of Kinanthropometry [32]. Waist was measured at the level of the umbilicus based on the protocol to assess metabolic syndrome in Japan (Japanese Society for the Study of Obesity 2016) [33], and 28 NWO and NWL measurements were referenced to the antecedent literature [34]. 

### 2.3. Body Composition

Eight-electrode multifrequency bioelectrical impedance analysis (MFBIA) was used for body composition measurements (The MC-780MA-N, TANITA Corp., Tokyo, Japan). All participants were instructed to wear light clothes with no socks and shoes. To ensure the accuracy of the BIA method, all the participants had body composition measured between 9 and 10 a.m. The participants did not eat or drink in the morning, and data collection began after voiding was performed. Data collection was done using the measurement guidelines from the device manual. Detailed information on contraindications, precautions, and specifications have been specified in the literature [35]. The measurement parameters were body weight, BMI, body fat mass, BFP, body muscle mass, body water mass, body water percentage, and visceral fat index.

### 2.4. Grouping

Based on BMI and BFP, all participants were assigned to either the NWL, NWO, or overweight and obese (OO group) groups. Participants with a BMI > 25 kg/m^2^ were automatically assigned to the OO group. Based on the results of a systematic review and meta-analysis on the definition of NWO and determination of adiposity, the recommended threshold value of NWO in Asians is a BFP of >20.6% in males and >33.4% in females [36]. Participants with a BMI < 25 kg/m^2^ were divided into the NWL and NWO groups based on their BFP in relation to the threshold percentages [37] (NWO—BFP > 20.6% in men and >33.4% in women; NWL—BFP < 20.6% in men and <33.4% in women).

### 2.5. Statistical Analysis

Descriptive data are presented as the mean ± standard deviation, median, or number and frequency. Normal data distribution was evaluated using the Shapiro–Wilk test. Homogeneity of variance was evaluated using the Levene’s test. To determine the significance of the differences, the Wilcoxon test was used for non-normally distributed data, and a one-way analysis of variance was used for normally distributed data. Differences in circumferences, circumference ratios, and body compositions of the NWL, NWO, and OO groups were analyzed using a one-way analysis of variance. Pearson or Spearman’s analyses were used to determine the correlation between the circumference ratios and BFP. Abnormal BFP was used as the dependent variable, and items that were statistically different between the NWO and NWL groups were used as independent variables. Odds ratios were calculated using binary logistic regression analysis after adjusting for age and sex. To avoid the effect of multicollinearity, variables with low correlation coefficients for BFP were excluded when the Pearson correlation coefficient between two circumferences or circumference ratios exceeded 0.7. A multicollinearity test was performed using the variance inflation factor. Receiver operating characteristic (ROC) curves were plotted to calculate the best circumference ratio cutoff value using the BFP definition of NWO. Sensitivity, specificity, and area under the ROC were calculated. Optimal cutoff values were defined as those that maximized the Youden index. Statistical analysis was performed using SPSS for Windows version 26.0 software (IBM Corp., Armonk, NY, USA). Statistical significance was set at *p* < 0.05.

## 3. Results

### 3.1. Basic Characteristics and Body Composition

The number of individuals with specific BMI range distributions is shown in Table 2. Among the participants, 47 (28.7%) were stratified into the NWL group, 30 (18.3%) into the NWO group, and 87 (53.0%) into the OO group. There were no statistical differences regarding age or height among the three groups; however, statistical differences in body weight, BMI, body fat mass, BFP, and visceral fat index were observed. Muscle mass and water mass did not differ between the NWL and NWO groups, body water percentage did not differ between the OO and NWO groups, and handgrip strength differed only between the OO and NWO groups. In the male group, only the visceral fat index did not differ between the NWL and NWO groups, the remaining results were consistent with the results of all participants analyses. The results of the female group analysis were all consistent with the analysis of all participants (Table 3).

### 3.2. Circumference and Circumference Ratio

In all participants, forearm, waist, hip, thigh, height/mid-upper arm, waist/height, hip/height, and (waist + hip)/height were statistically different among the three groups. Waist/hip, thigh/hip, thigh/height, and calf/waist ratios did not differ between the OO and NWO groups. The wrist, neck, calf, did not differ between the NWL and NWO groups. In the male group, waist, hip, hip/height, and (waist + hip)/height were statistically different among the three groups. Wrist, calf, waist/height and waist/hip ratios did not differ between the NWL and NWO groups, thigh and thigh/height ratios did not differ between the OO and NWO groups. In female group, mid-upper arm, waist, (waist + hip)/height, waist/height, hip/height and thigh/height were statistically different among the three groups. Wrist, neck, hip, thigh and calf did not differ between the NWL and NWO groups, waist/hip ratios did not differ between the OO and NWO groups (Table 3).

### 3.3. Correlation with BFP

Waist/height, BMI, thigh/height, hip/height, and (waist + hip)/height ratios were significantly correlated with BFP. In the male group, BMI, waist/height, (waist + hip)/height and waist/hip ratios were significantly correlated with BFP. In the female group, (waist + hip)/height, waist/height, thigh/height, hip/height, waist/hip ratios were significantly correlated with BFP (Table 4).

### 3.4. Binary Logistic Regression Analysis

We found no evidence of collinearity among the explanatory variables (mean-variance inflation factor of 3.04, range: 2.16–4.40). BMI, (waist + hip)/height, and thigh/height can be used as parameters for predicting abnormal BFP. (Waist + hip)/height, waist/height and waist/hip can be used as parameters for predicting abnormal BFP in males. (Waist + hip)/height, waist/height, waist/hip, hip/height, thigh/height and BMI can be used as parameters for predicting abnormal BFP in females (Table 5).

### 3.5. ROC Analysis and Cutoff Values

**Figure 1:**
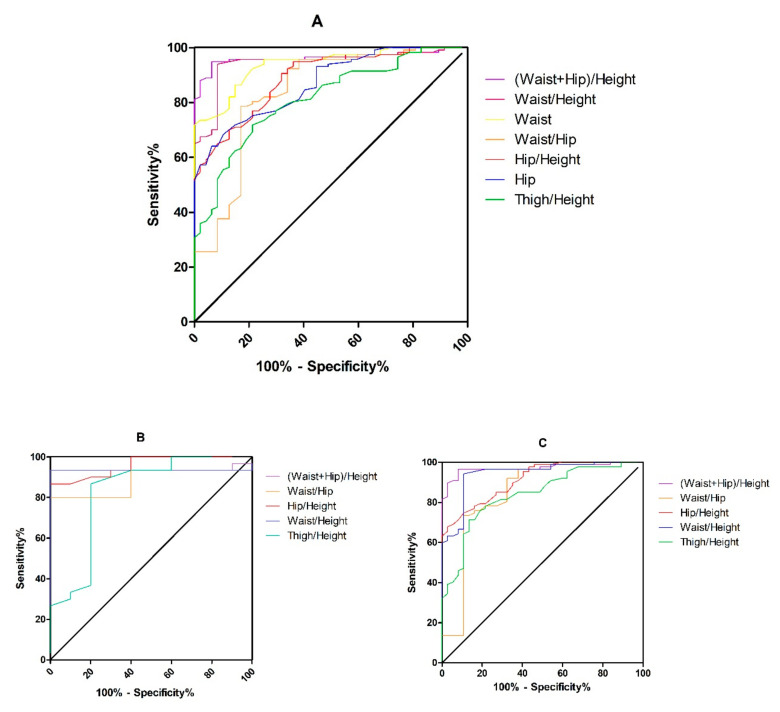
Comparison of ROC curves for body circumference or circumference ratio as independent risk factors. Abbreviation: ROC = receiver operating characteristic. (**A**): All participants. (**B**): Male group. (**C**): Female group.

Among all indicators, the largest Youden index for the prediction of excess BFP was for BMI (0.936) followed by (waist + hip)/height (0.885) ratio. The best circumference ratio for predicting BFP used waist, hip, and height measurements. Both male and female groups showed a consistent trend, the largest Youden index for the prediction of excess BFP was for (waist + hip)/height (0.885 or 0.833) ratio. An abnormally high BFP can be predicted when the (waist + hip)/height ratio exceeds 1.048 in males or 1.115 in females. To predict health risk in the Chinese population, the upper limit for a normal BMI should be lowered to 23.4 kg/m^2^ (Table 6, Figure 1). GraphPad Prism 5.0 (GraphPad Software, San Diego, CA, USA) was used to create the artwork.

## 4. Discussion

This study aimed to utilize simple anthropometric measurements to predict BFP. These simple and cost-effective measurements can be used anytime and anywhere. A better health risk assessment of metabolic diseases caused by abnormal body fat accumulation is highly relevant during the coronavirus disease pandemic, especially for individuals with NWO. To the best of our knowledge, this is the first study to investigate a screening indicator for health risk assessment in Chinese individuals with NWO.

Based on the results of the present study, it is clear that there are significant differences regarding BFP and body circumference between the NWL, NWO, and OO groups. Compared with BMI, the correlation between body circumference and BFP was higher. Thus, the (waist + hip)/height ratio can be used as an independent risk factor for predicting abnormal BFP and is indicated when it exceeds 1.115. We also suggest that the upper limit for a normal BMI for Chinese individuals bereduced to 23.4 kg/m^2^.

Studies have shown that compared with a BMI of 18.5–22.9 kg/m², a BMI of 23.0–24.9 kg/m² increases the risk for diabetes by 43% and 41% in men and women, respectively [38]. The optimal BMI threshold for diabetes screening is 23.8 kg/m² in Asia and 28.3 kg/m² in America. Therefore, BMI classification values should not be the same for all countries. BMI classifies more than half of individuals with an abnormal BFP as normal [39]. Therefore, we believe that the vast majority of these individuals are likely to be NWO, as the BMI of the NWO group in this study was 24.1 ± 0.7 kg/m², which is the upper limit of normal BMI.

Body circumferences are a good indicator of visceral fat [40]; thus, we should continue to perform anthropometric measurements after using the initial BMI screening to accurately assess BFP. We recommend utilizing (waist + hip)/height ratio, rather than the currently widely used WC or WHR. Furthermore, there is a significant difference in fat distribution between males and females [41]. Abdominal fat in males is 8–11% higher than that of females, while females’ hip fat is 6–9% higher than that of males. In postmenopausal females, estrogen levels decline and fat from the hips shift to the abdomen [42]. The fat distribution characteristics of postmenopausal females tend to be similar to that of males, leading to increased insulin resistance and cardiovascular disease risks [43]. Therefore, it is more accurate to predict obesity or health risk from the accumulation of body fat in the abdomen and hips as a whole [44].

According to the results of the ROC analysis, although (waist + hip)/height ratio best evaluates BFP among all the anthropometric measurements, BMI is an indicator that does not need to be completely replaced. We presume that the reason BMI showed better sensitivity and specificity for obesity is that we did not use a BMI of 24.9 kg/m² as the only differentiating factor, but also included whether the BFP was abnormal or not as the dependent variable. The individuals who were NWO were also classified into the abnormal BFP group, even though their BMI was not higher than 24.9 kg/m². We further suggest that the upper limit of the normal BMI value should be lowered to 23.4 kg/m² to better screen the NWO group.

In the results of analysis, both the male and female groups, were consistent with the results for the overall participants, all of which recommended (waist + hip)/height as the new brief index for obesity screening. ROC analysis in either the male group, female group, or all participants showed that the Youden index (sensitivity + specificity – 1) of (waist + hip)/height was the highest. Moreover, (waist + hip)/height was well correlated with body fat percentage in both male and female group. Although there were sex differences in basic physical measures (height, weight, BMI) and body circumference (waist circumference, hip circumference), the (waist + hip)/height was the circumferential diameter ratio after normalization for height. Additionally, we found that the data of (waist + hip)/height in this study was not statistically different between sexes (1.13 ± 0.08 in males and 1.15 ± 0.10 in females, *p* = 0.117, t = 1.574, 95% CI: −0.064–0.007). This implies that the indicator can be used for all sex groups and does not need to be differentiated for different sexes.

This study had some limitations. First, owing to coronavirus disease, only data from 164 participants were collected; more data from a larger sample size is required. Second, the participants were all from Beijing, and data from participants in other areas need to be collected. Third, the participants were mostly aged between 50 and 64 years; more detailed studies on specific age groups are needed. A small number of participants and geographical and age-related restrictions on the population of this study may limit the generalizability of the study’s findings to all Chinese populations. Therefore, large-scale studies with broader inclusion criteria are needed to validate results.

## 5. Conclusions

This study showed the physical characteristics of people with different BFPs using body composition evaluation and anthropometric measurements. It is recommended to use (waist + hip)/height ratio as a new and simple body fat assessment indicator for application in daily life to better screen for NWO and abnormal BFP. In the future, we recommend that the obesity and health risks assessment in the Chinese population apply the adjusted BMI (upper limit value of 23.4 kg/m²), and compute for the (waist + hip)/height ratio for a more comprehensive assessment. The combined assessment of anthropometric measurements and BMI allows for better screening of abnormal BFP, without the increased cost associated with BIA or other body composition assessment techniques. Presently, with the coronavirus disease pandemic, our method can be promoted and applied as an easy screening indicator for home use.

## Figures and Tables

**Table 1:** Anthropometric measurement methods used in the study and the corresponding percentage technical error of measurement (% TEM).

Anthropometric Measurement	Method	% TEM
wrist	The wrist girth is the minimum girth measurement perpendicular to the long axis of the forearm and distal to the ulnar styloid processes	1.06
upper arm	The maximum girth of the upper arm. The subject assumes a relaxed position with the arms hanging by the side of the body. The tape should be positioned perpendicular to the long axis of the humerus while the muscles of the arm are relaxed	1.16
neck	The circumference of the neck is measured immediately superior to the thyroid cartilage and perpendicular to the long axis of the neck	1.45
waist	The circumference of the waist at the level of umbilicus, perpendicular to the long axis of the trunk	1.39
hip	The circumference of the hip at the level of their greatest posterior protuberance, perpendicular to the long axis of the trunk	1.34
thigh	The girth of the thigh is taken 1 cm below the level of the gluteal fold, perpendicular to the long axis of the thigh	1.27
calf	The mid-calf circumference is defined as the maximum girth of the calf	1.09

% TEM: percentage technical error of measurement.

**Table 2:** BMI range of the participants.

BMI kg/m^2^	Male (%) *n* = 40	Female (%) *n* = 124
<18.5	0 (0%)	4 (3.2%)
18.5–25	16 (40%)	56 (45.2%)
25–30	22 (55%)	55 (44.4%)
>30	2 (5%)	9 (7.2%)

Male participants: maximum BMI 30.5 kg/m^2^, minimum 20.7 kg/m^2^; female prticipants: maximum BMI 34.1 kg/m^2^, minimum 17.5 kg/m^2.^

**Table 3:** Differentiation among the three groups.

	Normal-Weight Lean	Normal-Weight Obese	Overweight and Obesity	F	*p*
**All** **participants**	*n* = 47 (28.7%)	*n* = 30 (18.3%)	*n* = 87 (53.0%)		
Age (years)	56.2 ± 8.8	54.9 ± 8.4	55.5 ± 8.5	0.242	0.785
Height (cm)	163.8 ± 5.4	162.9 ± 6.2	164.2 ± 8.3	0.376	0.687
Weight (kg)	57.3 ± 6.4 ^#^*	63.9 ± 4.3 ^#^^⬙^	74.4 ± 9.3 *^⬙^	77.295	<0.001
Body mass index (kg/m^2^)	21.3 ± 1.5 ^#^*	24.1 ± 0.7 ^#^^⬙^	27.5 ± 1.9 *^⬙^	225.250	<0.001
Grip strength (kg)	26.7 ± 6.9	23.4 ± 4.9 ^⬙^	27.7 ± 7.4 ^⬙^	4.370	0.014
Body muscle mass (kg)	39.9 ± 6.5 *	40.3 ± 6.1 ^⬙^	44.8 ± 9.5 *^⬙^	7.042	<0.001
Body fat mass (kg)	14.8 ± 3.6 ^#^*	20.9 ± 2.4 ^#^^⬙^	26.9 ± 5.3 *^⬙^	117.011	<0.001
Body fat percentage (%)	26.0 ± 6.0 ^#^*	33.0 ± 5.3 ^#^^⬙^	36.7 ± 6.7 *^⬙^	44.966	<0.001
Body water mass (kg)	29.6 ± 5.1 *	30.5 ± 4.1 ^⬙^	34.9 ± 6.2 *^⬙^	16.240	<0.001
Body water percentage (%)	51.4 ± 4.8 ^#^*	47.6 ± 3.8 ^#^	46.6 ± 3.7 *	21.412	<0.001
Visceral fat index	6.0 ± 2.0 ^#^*	7.7 ± 1.6 ^#^^⬙^	10.5 ± 3.1 *^⬙^	49.244	<0.001
Mid-upper Arm (cm)	25.0 ± 3.9 ^#^*	28.7 ± 6.2 ^#^^⬙^	31.6 ± 3.6 *^⬙^	36.995	<0.001
Waist (cm)	76.6 ± 7.9 ^#^*	87.1 ± 7.4 ^#^^⬙^	94.9 ± 7.4 *^⬙^	90.937	<0.001
Hip (cm)	94.1 ± 4.7 ^#^*	97.7 ± 3.3 ^#^^⬙^	103.8 ± 5.2 *^⬙^	67.006	<0.001
Thigh (cm)	49.1 ± 5.6 ^#^*	53.4 ± 2.5 ^#^^⬙^	56.1 ± 5.6 *^⬙^	28.384	<0.001
Height/Mid-upper Arm	6.77 ± 1.45 ^#^*	6.00 ± 1.72 ^#^^⬙^	5.27 ± 0.69 *^⬙^	25.178	<0.001
Waist/Height	0.47 ± 0.05 ^#^*	0.54 ± 0.05 ^#^^⬙^	0.58 ± 0.04 *^⬙^	98.165	<0.001
Hip/Height	0.57 ± 0.02 ^#^*	0.60 ± 0.02 ^#^^⬙^	0.63 ± 0.04 *^⬙^	48.802	<0.001
(Waist + Hip)/Height	1.04 ± 0.06 ^#^*	1.14 ± 0.07 ^#^^⬙^	1.21 ± 0.07 *^⬙^	94.092	<0.001
Waist/Hip	0.81 ± 0.07 ^#^*	0.89 ± 0.06 ^#^	0.91 ± 0.05 *	42.401	<0.001
Thigh/Hip	0.52 ± 0.05 ^#^*	0.55 ± 0.02 ^#^	0.54 ± 0.04 *	4.578	0.012
Thigh/Height	0.30 ± 0.03 ^#^*	0.33 ± 0.02 ^#^	0.34 ± 0.04 *	26.596	<0.001
Calf/Waist	0.45 ± 0.05 ^#^*	0.40 ± 0.03 ^#^	0.40 ± 0.03 *	37.554	<0.001
Wrist (cm)	15.3 ± 0.9 *	15.4 ± 1.5 ^⬙^	16.8 ± 1.5 *^⬙^	21.731	<0.001
Neck (cm)	33.6 ± 2.9 *	34.8 ± 5.3 ^⬙^	36.9 ± 3.8 *^⬙^	11.449	<0.001
Calf (cm)	34.2 ± 2.7 *	35.1 ± 2.4 ^⬙^	37.7 ± 3.0 *^⬙^	26.336	<0.001
**Male group**	*n* = 10 (25%)	*n* = 6 (15%)	*n* = 24 (60%)		
Age (years)	55.7 ± 10.1 ^#^	44.2 ± 2.6 ^#^	50.0 ± 8.5	3.635	0.036
Height (cm)	169.0 ± 4.7 *	173.8 ± 1.6	175.0 ± 5.3 *	5.573	0.008
Weight (kg)	63.8 ± 6.3 ^#^*	71.7 ± 3.4 ^#^^⬙^	84.9 ± 4.5 *^⬙^	71.299	<0.001
Body mass index (kg/m^2^)	22.3 ± 1.0 ^#^*	23.7 ± 0.7 ^#^^⬙^	27.7 ± 1.0 *^⬙^	132.274	<0.001
Grip strength (kg)	37.7 ± 3.4 ^#^	29.4 ± 4.1 ^#^^⬙^	37.2 ± 4.9 ^⬙^	8.153	0.001
Body muscle mass (kg)	50.7 ± 4.6 *	51.9 ± 2.3 ^⬙^	58.7 ± 5.5 *^⬙^	11.240	<0.001
Body fat mass (kg)	10.0 ± 1.9 ^#^*	16.4 ± 1.3 ^#^^⬙^	23.9 ± 5.6 *^⬙^	34.997	<0.001
Body fat percentage (%)	15.6 ± 1.8 ^#^*	22.9 ± 0.8 ^#^^⬙^	28.9 ± 7.4 *^⬙^	18.311	<0.001
Body water mass (kg)	37.9 ± 4.1 *	38.3 ± 1.0 ^⬙^	43.3 ± 3.3 *^⬙^	12.320	<0.001
Body water percentage (%)	59.4 ± 2.9 ^#^*	53.6 ± 4.0 ^#^	50.8 ± 3.9 *	23.346	<0.001
Visceral fat index	9.0 ± 0.8 *	9.4 ± 3.1 ^⬙^	14.8 ± 2.0 *^⬙^	38.066	<0.001
Wrist (cm)	16.0 ± 0.8 *	16.9 ± 1.4 ^⬙^	18.1 ± 0.9 *^⬙^	17.799	<0.001
Mid-upper Arm (cm)	27.8 ± 1.7 ^#^	34.6 ± 5.8 ^#^^⬙^	30.0 ± 2.3 ^⬙^	10.345	<0.001
Neck (cm)	37.1 ± 2.2^#^	42.4 ± 6.3 ^#^	40.2 ± 2.8	5.232	0.010
Waist (cm)	93.1 ± 2.6 ^#^*	100.3 ± 3.7 ^#^^⬙^	105.6 ± 2.7 *^⬙^	82.974	<0.001
Hip (cm)	79.0 ± 6.0 ^#^*	86.3 ± 10.1 ^#^^⬙^	102.0 ± 2.0 *^⬙^	67.397	<0.001
Thigh (cm)	45.6 ± 6.6 ^#^*	55.9 ± 6.6 ^#^	56.1 ± 6.7 *	10.629	<0.001
Calf (cm)	36.2 ± 2.2 *	37.1 ± 1.5 ^⬙^	40.1 ± 2.1 *^⬙^	15.312	<0.001
(Waist + Hip)/Height	1.02 ± 0.02 ^#^*	1.07 ± 0.07 ^#^^⬙^	1.19 ± 0.04 *^⬙^	69.640	<0.001
Hip/Height	0.55 ± 0.02 ^#^*	0.58 ± 0.02 ^#^^⬙^	0.60 ± 0.02 *^⬙^	29.452	<0.001
Thigh/Height	0.27 ± 0.04 ^#^*	0.32 ± 0.01 ^#^	0.33 ± 0.04 *	8.485	0.001
Waist/Hip	0.85 ± 0.07 *	0.86 ± 0.07 ^⬙^	0.97 ± 0.02 *^⬙^	32.324	<0.001
Waist/Height	0.47 ± 0.03 *	0.50 ± 0.05 ^⬙^	0.58 ± 0.02 *^⬙^	72.095	<0.001
**Female group**	*n* = 37 (29.8%)	*n* = 24 (19.4%)	*n* = 63 (50.8%)		
Age (years)	56.4 ± 8.6	57.5 ± 7.2	57.6 ± 7.5	0.328	0.721
Height (cm)	162.4 ± 4.8 *	160.2 ± 3.0	160.1 ± 4.7 *	3.446	0.035
Weight (kg)	55.6 ± 5.3 ^#^*	61.9 ± 1.2 ^#^^⬙^	70.4 ± 7.2 *^⬙^	74.604	<0.001
Body mass index (kg/m2)	21.1 ± 1.5 ^#^*	24.2 ± 0.7 ^#^^⬙^	27.5 ± 2.2 *^⬙^	151.568	<0.001
Grip strength (kg)	23.7 ± 3.8	21.9 ± 3.9	24.1 ± 4.2	2.489	0.087
Body muscle mass (kg)	36.9 ± 2.6 *	37.4 ± 1.3 ^⬙^	39.5 ± 3.2 *^⬙^	12.038	<0.001
Body fat mass (kg)	16.1 ± 2.8 ^#^*	22.0 ± 0.8 ^#^^⬙^	28.1 ± 4.7 *^⬙^	124.723	<0.001
Body fat percentage (%)	28.7 ± 2.7 ^#^*	35.5 ± 1.5 ^#^^⬙^	39.7 ± 3.1 *^⬙^	187.913	<0.001
Body water mass (kg)	27.4 ± 2.2 *	28.6 ± 1.2 ^⬙^	31.6 ± 3.2 *^⬙^	34.037	<0.001
Body water percentage (%)	49.2 ± 2.2 ^#^*	46.1 ± 1.7 ^#^	45.0 ± 2.9 *	42.940	<0.001
Visceral fat index	5.1 ± 0.9 ^#^*	7.3 ± 0.8 ^#^^⬙^	7.4 ± 1.9 *^⬙^	150.835	<0.001
Mid-upper Arm (cm)	24.2 ± 4.0 ^#^*	27.3 ± 5.5 ^#^^⬙^	32.2 ± 3.8 *^⬙^	43.650	<0.001
Waist (cm)	76.0 ± 8.3 ^#^*	87.4 ± 6.8 ^#^^⬙^	92.2 ± 6.8 *^⬙^	58.250	<0.001
Wrist (cm)	15.1 ± 0.8 *	15.1 ± 1.4 ^⬙^	16.3 ± 1.4 *^⬙^	13.779	<0.001
Neck (cm)	32.7 ± 2.3 *	32.9 ± 2.9 ^⬙^	35.7 ± 3.4 *^⬙^	14.195	<0.001
Hip (cm)	94.4 ± 5.1 *	97.1 ± 2.8 ^⬙^	103.1 ± 5.7 *^⬙^	36.617	<0.001
Thigh (cm)	50.0 ± 5.0 *	52.8 ± 2.4 ^⬙^	56.1 ± 5.1 *^⬙^	20.253	<0.001
Calf (cm)	33.6 ± 2.6 *	34.6 ± 2.4 ^⬙^	36.7 ± 2.7 *^⬙^	17.490	<0.001
(Waist + Hip)/Height	1.05 ± 0.06 ^#^*	1.15 ± 0.06 ^#^^⬙^	1.22 ± 0.08 *^⬙^	65.686	<0.001
Waist/Height	0.47 ± 0.05 ^#^*	0.55 ± 0.05 ^#^^⬙^	0.58 ± 0.05 *^⬙^	63.144	<0.001
Hip/Height	0.58 ± 0.02 ^#^*	0.61 ± 0.02 ^#^^⬙^	0.65 ± 0.04 *^⬙^	46.909	<0.001
Thigh/Height	0.31 ± 0.03 ^#^*	0.33 ± 0.02 ^#^^⬙^	0.35 ± 0.03 *^⬙^	26.803	<0.001
Waist/Hip	0.80 ± 0.07 ^#^*	0.89 ± 0.06 ^#^	0.90 ± 0.05 *	32.474	<0.001

^#^ Statistical difference between NWL and NWO, * statistical difference between NWL and OO, ^⬙^ statistical difference between NWO and OO.

**Table 4:** Correlation of significant anthropometrical indicators with body fat percentage (*p* < 0.001).

	Waist/Height	BMI	Thigh/Height	Hip/Height	(Waist + Hip)/Height
**All participants**					
r	0.556	0.619	0.639	0.646	0.668
**Male group**					
	BMI	Waist/Height	(Waist + Hip)/Height	Waist/Hip	
r	0.684	0.617	0.594	0.593	
**Female group**					
	(Waist + Hip)/Height	Waist/Height	Thigh/Height	Hip/Height	Waist/Hip
r	0.806	0.781	0.753	0.739	0.613

Abbreviations: BMI = body mass index.

**Table 5:** Binary logistic regression analysis of risk factors for excess body fat percentage.

	OR	95% CI	*p*
**All participants**			
BMI	3.130	3.954–27.616	0.001
(Waist + hip)/height	5.205	3.004–9.018	0.013
Thigh/height	8.121	2.413–2.733	0.008
**Male group**			
Waist/height	1.208	1.037–1.407	0.001
(Waist + hip)/height	4.174	1.826–2.991	0.001
Waist/hip	4.894	1.409–1.699	0.002
**Female group**			
(Waist + hip)/height	8.059	2.407–2.698	0.001
Waist/hip	5.439	5.317–5.564	0.001
Hip/height	3.665	2.737–4.909	0.001
Waist/height	1.731	1.460–2.052	0.001
Thigh/height	1.216	2.503–5.907	0.001
BMI	2.543	3.824–6.910	0.001

Abbreviations: BMI = body mass index; CI = confidence interval; OR = odds ratio.

**Table 6:** ROC analysis of diagnostic indicators.

	AUC	Sensitivity (%)	Specificity (%)	Youden Index	Cutoff	*p*	95% CI
**All participants**							
BMI	0.992	95.7	97.9	0.936	23.369	<0.001	0.984–0.999
(Waist + hip)/height	0.964	94.9	93.6	0.885	1.115	<0.001	0.936–0.992
Waist/height	0.944	94.0	91.5	0.855	0.512	<0.001	0.908–0.980
Waist	0.938	73.5	97.9	0.714	88.25	<0.001	0.904–0.973
Waist/hip	0.837	78.6	83.0	0.616	0.869	<0.001	0.763–0.912
Hip/height	0.888	94.9	63.8	0.587	0.582	<0.001	0.838–0.939
Hip	0.869	68.4	89.4	0.578	99.25	<0.001	0.816–0.923
Thigh/height	0.812	71.8	78.1	0.499	0.323	0.035	0.744–0.880
**Male group**							
(Waist + hip)/height	0.937	93.3	90	0.833	1.048	0.001	0.851–0.999
Waist/hip	0.880	80	90	0.700	0.932	0.001	0.773–0.987
Hip/height	0.957	86.7	90	0.767	0.576	0.001	0.900–0.998
Waist/height	0.933	93.3	90	0.833	0.496	0.001	0.844–0.997
Thigh/height	0.835	90	80	0.700	0.276	0.002	0.665–0.998
**Female group**							
(Waist + hip)/height	0.971	96.6	91.9	0.885	1.115	0.001	0.944–0.998
Waist/hip	0.845	73.6	89.2	0.628	0.869	0.001	0.755–0.934
Hip/height	0.914	77	89.2	0.662	0.604	0.001	0.866–0.962
Waist/height	0.942	94.3	89.2	0.835	0.512	0.001	0.899–0.985
Thigh/height	0.836	71.3	86.5	0.578	0.329	0.001	0.762–0.980

Abbreviations: AUC = area under the receiver operating characteristic curve; BMI = body mass index; CI = confidence interval.

## Data Availability

Data are contained within the article.
